# Tree thinning as an option to increase herbaceous yield of an encroached semi-arid savanna in South Africa

**DOI:** 10.1186/1472-6785-5-4

**Published:** 2005-05-28

**Authors:** Gert N Smit

**Affiliations:** 1 Department of Animal, Wildlife and Grassland Sciences, University of the Free State, P.O. Box 339, Bloemfontein 9300, Republic of South Africa

## Abstract

**Background:**

The investigation was conducted in a savanna area covered by what was considered an undesirably dense stand of *Colophospermum mopane *trees, mainly because such a dense stand of trees often results in the suppression of herbaceous plants. The objectives of this study were to determine the influence of intensity of tree thinning on the dry matter yield of herbaceous plants (notably grasses) and to investigate differences in herbaceous species composition between defined subhabitats (under tree canopies, between tree canopies and where trees have been removed). Seven plots (65 × 180 m) were subjected to different intensities of tree thinning, ranging from a totally cleared plot (0 %) to plots thinned to the equivalent of 10 %, 20%, 35 %, 50% and 75 % of the leaf biomass of a control plot (100 %) with a tree density of 2711 plants ha^-1^. The establishment of herbaceous plants (grasses and forbs) in response to reduced competition from the woody plants was measured during three full growing seasons following the thinning treatments.

**Results:**

The grass component reacted positively to the tree thinning in terms of total dry matter (DM) yield, but forbs were negatively influenced. Rainfall interacted with tree density and the differences between grass DM yields in thinned plots during years of below average rainfall were substantially higher than those of the control. At high tree densities, yields differed little between seasons of varying rainfall. The relation between grass DM yield and tree biomass was curvilinear, best described by the exponential regression equation. Subhabitat differentiation by *C. mopane *trees did provide some qualitative benefits, with certain desirable grass species showing a preference for the subhabitat under tree canopies.

**Conclusion:**

While it can be concluded from this study that high tree densities suppress herbaceous production, the decision to clear/thin the *C. mopane *trees should include additional considerations. Thinning of *C. mopane *with the exclusive objective of increasing productivity of the grass layer would thus invariably involve a compromise situation where some trees should be left for the sake of the qualitative benefits on the herbaceous layer, soil enrichment, provision of browse and stability of the ecosystem.

## Background

In addition to browsing by domestic stock and game the direct uses of woody plants in southern African savannas include their use as firewood, rough construction timber, the production of charcoal and woodcarvings. In areas where trees are used for timber, harvest rates commonly exceed replacement rates. In other areas where woody plants are not subjected to harvesting and where cattle and game ranching are practised exclusively, an increase in woody plant abundance is common. This increase in woody plant abundance is commonly referred to as "bush encroachment" and involves the invasion of grasslands and the thickening of savanna [1].

The reasons for bush encroachment in savanna are diverse and complex. In most situations the determinants of savanna ecosystems were modified by man, either directly or indirectly. These determinants may either be primary (such as climate and soil) or secondary (such as fire and the impact of herbivores) [2-4]. The latter are of particular interest since, although they act within the constraints imposed by the primary determinants, they can often be directly modified by management. Examples are the exclusion of occasional hot, top killing brush fires, the replacement of most of the indigenous browsers and grazers by domestic (largely grazing) livestock often at extremely high stocking rates, the restriction of movement of herbivores by the erection of fences, long-term overgrazing of the herbaceous layer, notably during wet seasons, and the provision of artificial watering points [5,6].

In extensively managed semi-arid savannas, the productivity of herbaceous plants, notably the grasses, is of primary importance. The *Colophospermum mopane *Kirk ex J. Léonard (Kirk ex Benth) dominated savanna of South Africa, like most of the southern African savanna ecosystems, is water-limited and an increase in woody plant abundance invariably results in the suppression of herbaceous plants (e.g. [7-11]). Due to this suppression effect, the grazing capacity of large areas of the South African *C. mopane *savanna is reported to have declined due to bush encroachment, often to such an extent that many previously economic livestock properties are now no longer economic [6]. This is often the major reason why tree thinning or even total clearing is considered. The most commonly used methods of bush control include both mechanical and chemical measures. Arboricides with tebuthiuron as active ingredient are often used, but due to the non-selective nature of this arboricide, trees are also mechanically cut and the stumps treated with arboricides of which picloram is the most common active ingredient. Due to a general lack of adequate herbaceous biomass for fuel, fire is not used in the area.

Results of tree thinning may, however, differ between vegetation types, and is complicated by the existence of not only negative tree-grass interactions, but also positive tree-grass interactions. Due to the enrichment of soil under tree canopies [12-14], trees may have positive effects on grass growth. Positive interactions, such as the association of certain desirable grass species (notably *Panicum maximum*) with tree canopies, are a consequence of subhabitat differentiation (canopied and uncanopied subhabitats). Subhabitat differentiation is dependent on tree density, tree species and tree size [14,15], while interactions with soil can also play a role.

The *C. mopane *savanna is an important savanna vegetation type of southern Africa (South Africa, Namibia, Botswana and Mozambique) and the total area in southern Africa under *C. mopane *vegetation types is estimated at 555000 km^2 ^[16]. The *C. mopane *trees have extensive root systems [17] and their selective removal has a profound effect on the soil water regime [18]. This will invariably influence the tree-grass competitive interaction. An understanding of the exact nature and magnitude of such influences is an important prerequisite towards an understanding of the complex biological interactions that exist in these ecosystems.

As part of a comprehensive investigation into the effect of tree thinning on the South African *C. mopane *savanna, the objectives of this study were: (i) to determine the influence of intensity of tree thinning on the dry matter yield of herbaceous plants (notably grasses), (ii) to establish relations between tree density and herbaceous production, and (iii) to investigate differences in herbaceous species composition between defined subhabitats (under tree canopies, between tree canopies and where trees have been removed).

### Study area

The study was conducted in the Limpopo Province of South Africa on a site located at 29°12'E, 22°19'S, 560 m above sea level. The savanna vegetation is locally described by Acocks [19] as "Mopani veld" and by Low & Rebelo [20] as "Mopane Bushveld". Louw [21] made a further division of seven plant communities within the South African Mopane Bushveld, and the study area was located in, what he named the *Colophospermum-Boscia *community. This community covers about 60 000 ha of the Mopane Bushveld. Louw [21] described this community as a virtually pure stand of *Colophospermum mopane *(synonym: *Hardwickia mopane*), interposed with a few individuals of *Boscia foetida *subsp. *rehmanniana *and *Salvadora australis (*synonym: *S. angustifolia *var. *australis*). Within the study area the most important grass species are *Enneapogon cenchroides, Aristida adscensionis*, *Brachiaria deflexa*, *Cenchrus ciliaris *and *Digitaria eriantha*.

The study area, before tree thinning, was characterized by the virtual absence of herbaceous plants, accompanied by severe soil degradation in the form of surface erosion and crust formations. Crust formations are known to reduce infiltration and cause substantial losses due to rainfall runoff (e.g. [22-25]). The site was previously used as grazing for cattle, but due to a lack of adequate grazing, the cattle was removed from the area for a period of at least five years. Since then the area has mainly been grazed and browsed by an unknown number of free ranging game species.

The rainy season usually extends from October to March inclusively, but rainfall is irregularly distributed and unpredictable. Mean long-term seasonal rainfall (July-June) for the period 1966/67 to 1990/91 was 376 mm (SE ± 27.6, range: 140–620 mm). The probability of rain falling during January is greater than for other months. The area is largely frost-free and is well known for its high summer temperatures and moderate to warm winter temperatures.

The underlying rock type is mainly sandstone [21] and the soil is predominantly sandy (80% sand, 8 % silt, 12 % clay). A detailed description of the soil is given by Smit & Rethman [18].

## Results

### Areas of subhabitats

Three subhabitats were distinguished: between tree canopies (uncanopied – UCA), under tree canopies (canopied – CA) and where trees have been removed (removed canopy – RCA). The areas covered by the various subhabitats are presented in Table 1. The subhabitat between trees (uncanopied – UCA) predominates, also with small variation between treatments (mean of 85.8 %). Through tree thinning the subhabitat under trees canopies (canopied – CA) decreased over the gradient of decreasing tree density, up to the point of no representation in the 0% plot. In contrast, the subhabitat where trees were removed (removed canopy – RCA), increased over this gradient, not being represented in the control plot (100 % plot).

**Table 1:** Percentages of the total surface area covered by the various habitats and subhabitats in each of the experimental plots.

Subhabitat	Experimental plot	Area (%)
Between trees	0 %	87.10
,,	10 %	86.58
,,	20 %	83.99
,,	35 %	86.65
,,	50 %	87.46
,,	75 %	85.92
,,	100 %	82.92
		
Under trees	0 %	0.00
,,	10 %	2.85
,,	20 %	5.57
,,	35 %	5.80
,,	50 %	8.82
,,	75 %	10.85
,,	100 %	17.08
		
Where trees	0 %	12.90
were removed	10 %	10.57
,,	20 %	10.44
,,	35 %	7.55
,,	50 %	3.72
,,	75 %	3.23
,,	100 %	0.00

### Leaf volume of the woody layer

The estimated number of Evapotranspiration Tree Equivalents (ETTE) ha^-1 ^of the *C. mopane *trees over the trial period is presented in Table 2. Detailed results and a discussion of the woody layer are reported elsewhere [26-28].

**Table 2:** The number of EvapotranspirationTree Equivalents (ETTE) ha^-1 ^within the various tree thinning plots and seasons.

Season	Exp. plot	ETTE ha^-1^
After thinning	10	605.5
	20	1 406.1
	35	1 717.4
	50	3 176.2
	75	3 376.7
	100	5 509.9
		
Season 1	10	707.5
	20	1 669.5
	35	2 008.0
	50	3 487.6
	75	3 602.9
	100	5 900.8
		
Season 2	10	809.9
	20	1 885.5
	35	2 136.1
	50	3 503.3
	75	3 769.3
	100	5 962.3
		
Season 3	10	998.4
	20	2 171.8
	35	2 540.8
	50	3 870.8
	75	4 197.2
	100	6 733.0

### Dry matter yield of the herbaceous layer

**Figure 1:**
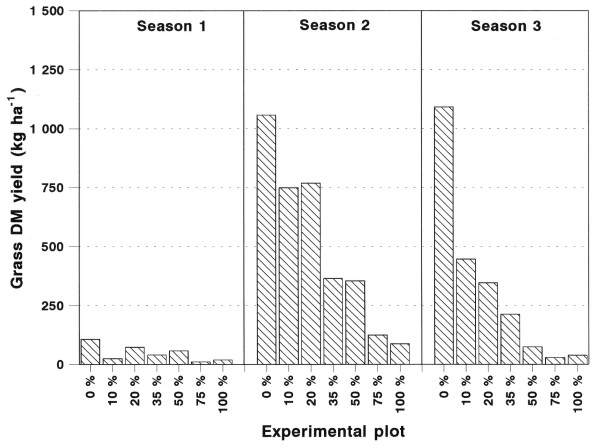
Total seasonal DM yields of grasses as measured in die various tree thinning treatment plots during the three seasons following the tree thinning (the DM yields of the uncanopied, canopied and removed canopy subhabitats were combined).

**Figure 2:**
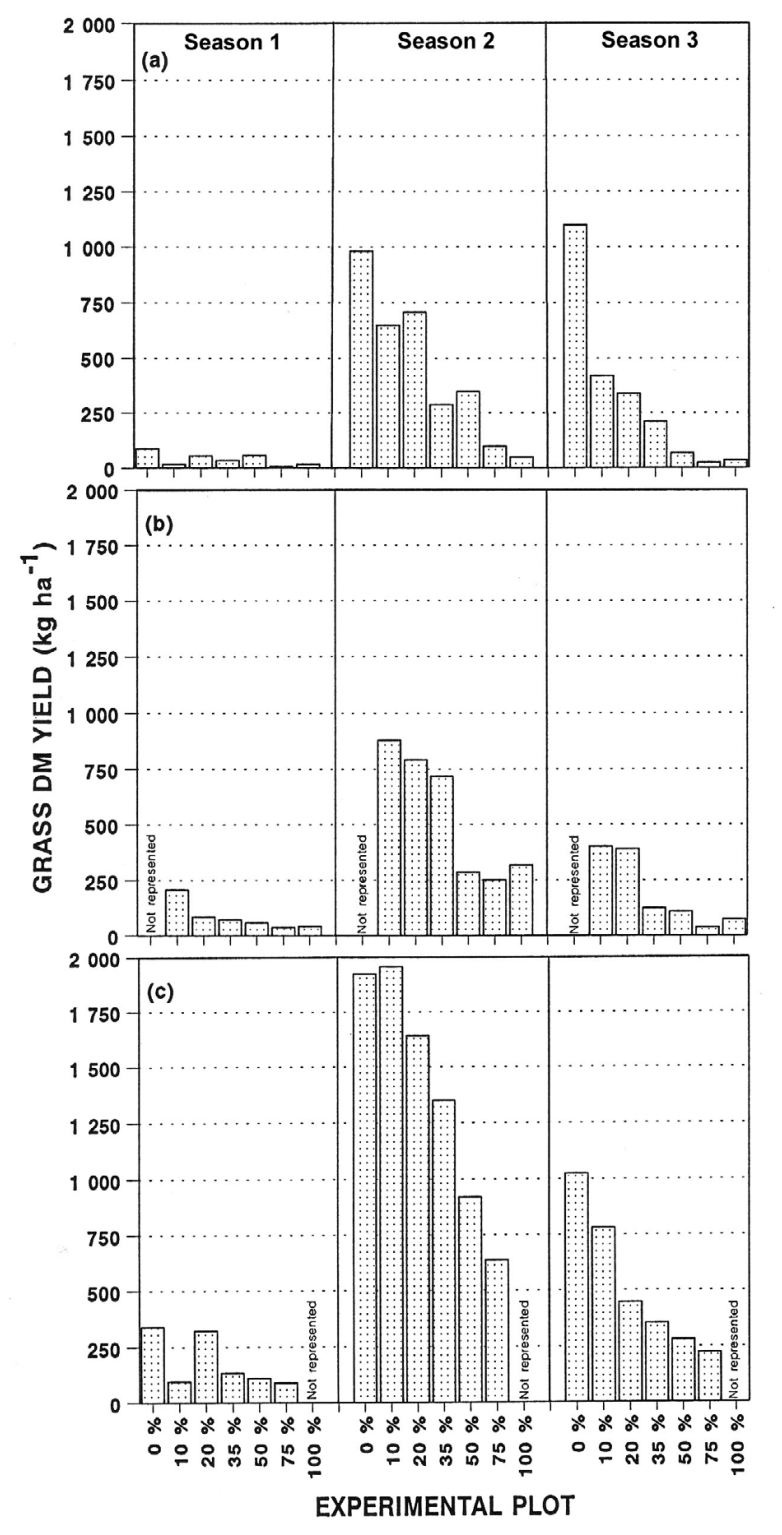
Total seasonal DM yields of grasses within the defined subhabitats during the three seasons following the tree thinning: (a) between tree canopies (uncanopied), (b) under trees (canopied), and (c) where trees have been removed (removed canopy).

**Figure 3:**
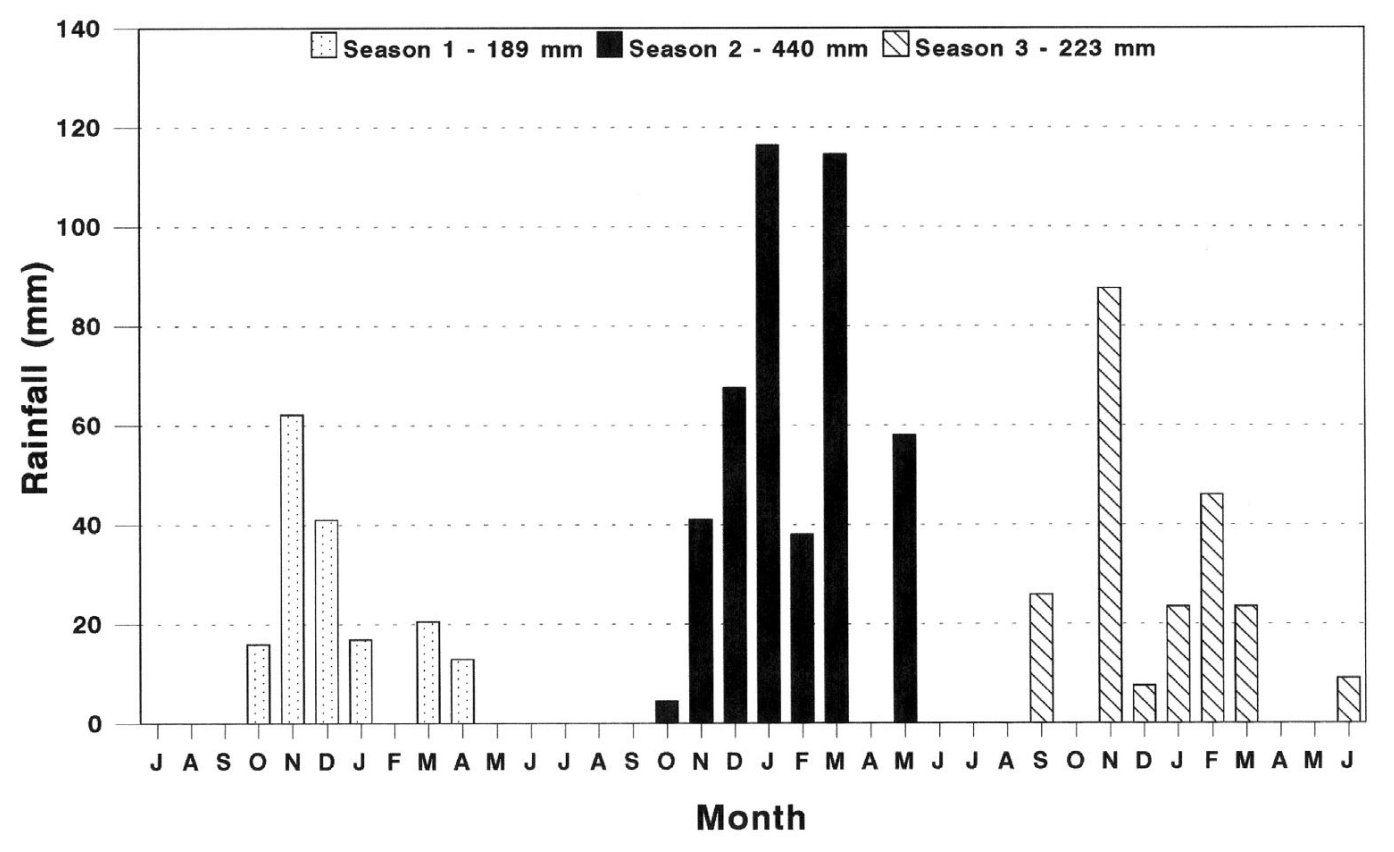
Monthly rainfall recorded at the *Colophospermum mopane *experimental site during the three seasons (July-June) of the trial period.

The total seasonal DM yield of grasses (subhabitats combined) is presented in Figure 1 and the total seasonal DM yield of grasses within the various subhabitats is presented in Figure 2. The grass DM yields were generally low during the first, low rainfall season season 1). In the following seasons the yields were substantially higher, with marked differences between treatments. Comparison of the grass DM yields between subhabitats, revealed differences. The yields between tree canopies (UCA) (Figure 2a) were initially of the same order as under tree canopies (CA) (Figure 2b), with the yields where trees have been removed (RCA) the highest (Figure 2c). While the seasonal grass yield patterns largely followed the rainfall pattern (Figure 3), the yields of the UCA subhabitat in the totally cleared plot (0% plot) continued to improve during the third season, which received less than half the rainfall of the previous season (season 2).

The fitting of polynomials to data of season 1 was unsuccessful, mainly due to the low rainfall and subsequent poor response of the grass layer. Polynomials were subsequently only fitted to the data for seasons 2 and 3 (Table 3). The testing of paired combinations (contrasts) [29] of these selected polynomials for significant differences on the x-axis (ETTE ha^-1^) showed that total grass DM yields did not differ significantly (P > 0.05) between the UCA and CA-subhabitats, during both seasons 2 and 3. In contrast, during season 2, up to 3927 ETTE ha^-1^, grass DM yields differed significantly (P < 0.05) between the UCA and the RCA-subhabitats, with the yields being higher in the RCA-subhabitat. These differences changed during season 3, with yields not differing significantly (P > 0.05) between the latter subhabitats over the complete ETTE gradient. The test of the contrast CA versus RCA showed that during season 2 yields differed significantly (P < 0.05) up to a density of 3817 ETTE ha^-1 ^(0 %, 10%, 20 %, 35 % and 50 % plots), with the yields being higher in the RCA-subhabitat. Similar to the UCA/RCA contrast, yields did not differ significantly (P > 0.05) between these subhabitats during season 3.

**Table 3:** Polynomials with the best fit (y = total grass DM yield, x = ETTE ha^-1^)

Subhabitat	Season after thinning	Polynomial	r^2^	P
UCA	2	quadratic: y = 948.0 - 0.278 + 0.000021x^2^	0.78	0.021
UCA	3	quadratic: y = 980.0 - 0.3985 + 0.000039x^2^	0.90	0.005
CA	2	cubic: 686.3 - 0.4284x + 0.000254x^2 ^+ 0.29E-7x^3^	0.99	0.003
CA	3	quadratic: 630.0 - 0.2132x + 0.000019x^2^	0.64	0.099
RCA	2	quadratic: 1 968.0 - 0.066x - 0.000073x^2^	0.95	0.005
RCA	3	quadratic: 1 047.3 - 0.3558x + 0.000038x^2^	0.98	0.001

### Relationship between tree leaf biomass and herbaceous dry matter yield

**Figure 4:**
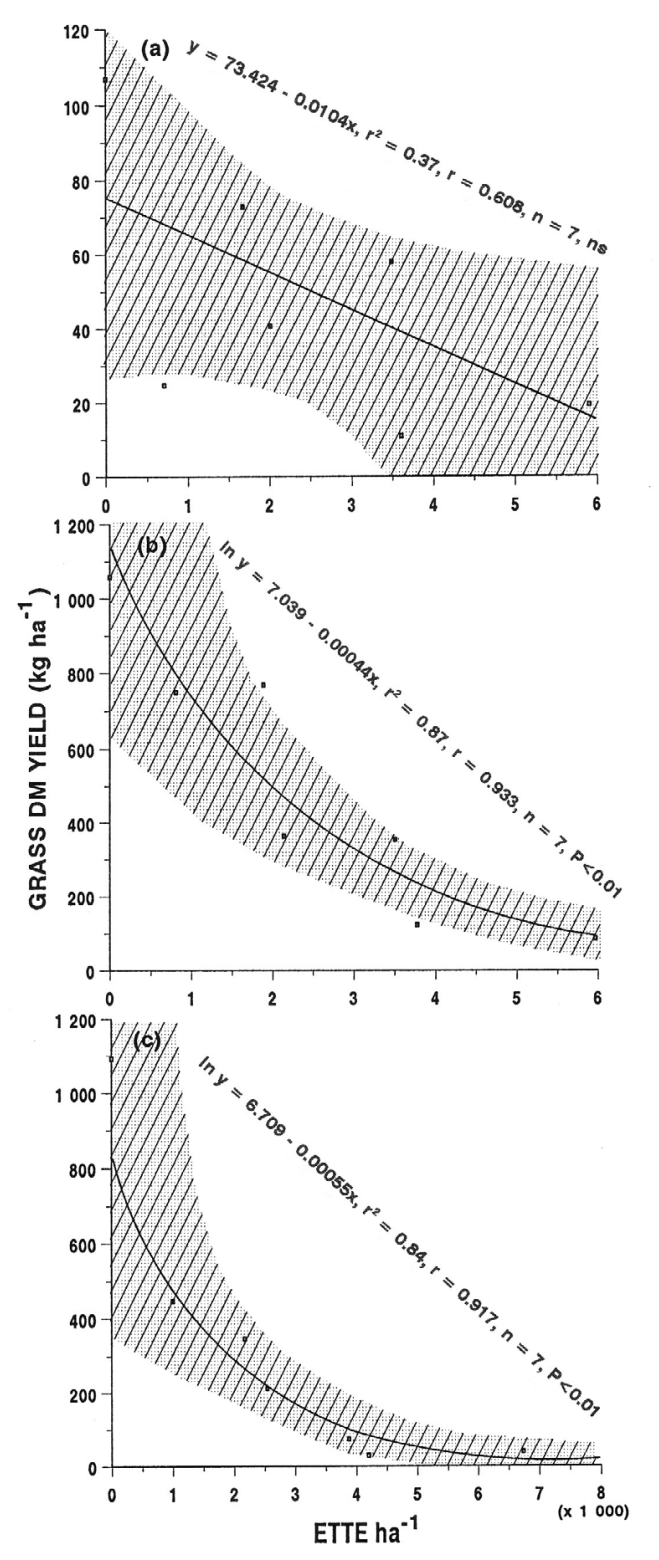
Results of the regression analyses of the relations between the grass DM yield (subhabitats combined) and the Evapotranspiration Tree Equivalents (ETTE) ha^-1 ^(shaded area shows the 95 % confidence limits): (a) season 1, (b) season 2, and (c) season 3.

The relationship between tree leaf biomass, expressed as Evapotranspiration Tree Equivalents (ETTE) ha^-1 ^and total grass DM yield (all subhabitats combined) of each treatment plot was established (Figure 4). The relationships between ETTE ha^-1 ^and grass DM yield within each of the defined subhabitats are presented in Table 4. The relations between ETTE ha^-1 ^and the DM yield of forbs within these subhabitats are presented in Table 5.

**Table 4:** Results of the regression analyses of the relations between the DM yields of grasses within the defined subhabitats (dependent variable) and Evapotranspiration Tree Equivalents (ETTE) ha^-1 ^(independent variable).

Subhabitat	Season	Regression equation	r^2^	r	n	P
Between trees (UCA)	1	y = 57.188 - 0.00765x	0.282	-0.532	7	0.220 ns
	2	ln y = 7.017 - 0.000510x	0.861	-0.928	7	0.003 **
	3	ln y = 6.708 - 0.000579x	0.828	-0.910	7	0.004 **
						
Under trees (CA)	1	ln y = 5.052 - 0.000274x	0.679	-0.824	7	0.044 *
	2	ln y = 6.936 - 0.000257x	0.662	-0.814	7	0.049 *
	3	ln y = 6.099 - 0.000349x	0.567	-0.753	7	0.084 ns
						
Trees removed (RCA)	1	y = 274.648 - 0.0479x	0.358	-0.599	7	0.209 ns
	2	ln y = 7.745 - 0.000284x	0.868	-0.932	7	0.007 **
	3	ln y = 6.936 - 0.000466x	0.980	-0.990	7	0.002 **

**Table 5:** Results of the regression analyses of the relations between the DM yields of forbs within the defined subhabitats (dependent variable) and Evapotranspiration Tree Equivalents (ETTE) ha^-1 ^(independent variable).

Subhabitat	Season	Regression equation	r^2^	r	n	P
Between trees (UCA)	1	ln y = 2.756 + 0.000214x	0.137	0.370	7	0.414 ns
	2	y = 43.396 + 0.01467x	0.272	0.521	7	0.230 ns
	3	ln y = 3.911 + 0.000156x	0.126	0.355	7	0.434 ns
						
Under trees (CA)	1	y = - 65.59 + 0.12309x	0.675	0.822	7	0.045 *
	2	y = 90.378 + 0.05286x	0.813	0.902	7	0.014 *
	3	y = 139.76 + 0.04719x	0.416	0.645	7	0.167 ns
						
Trees removed (RCA)	1	y = 21.291 + 0.00130x	0.021	0.146	7	0.783 ns
	2	y = 72.022 - 0.01599x	0.537	-0.733	7	0.098 ns
	3	ln y = 0.888 + 0.000824x	0.611	0.781	7	0.066 ns

From Figure 4a there is a negative trend between ETTE ha^-1 ^and total grass DM yield of the combined subhabitats. However, following thinning, this negative trend changed significantly with each season. It changed from a non-significant (P > 0.05) linear relation during the first (dry) season (Figure 4a) to a significant curvilinear (P < 0.05) relationship during the second and third seasons (Figure 4b &4c). The best fit to these curvilinear relations was achieved by the exponential regression equation. The gradient of the curve was steeper in the relationship established for season 3 (Figure 4c), indicating an increasing difference between grass DM yields of the totally cleared plot (0 %) and the rest of the treatments.

Examination of the grass DM yields within the respective subhabitats revealed trends similar to that already presented for the combined subhabitats. Significant (P < 0.05) negative relationships between grass yield and ETTE ha^-1 ^are particularly eminent after the second and third seasons within the UCA and RCA-subhabitats. This negative relationship was less strongly defined in the CA-subhabitat (Table 4).

The reaction of forbs to the thinning of *C. mopane *differed markedly from that of the grasses (Table 5). With few exceptions, the yields of forbs were mostly positively associated with ETTE ha^-1^, though the relations were mostly statistically non-significant (P > 0.05). This implies that they were predominantly negatively affected by tree thinning. Some variation between subhabitats was also found, but a consistent pattern was lacking.

### Grass species differences between subhabitats

The mean percentage contributions (on a dry mass basis) of the most abundant grass species to the total grass DM yield within the defined subhabitats are presented in Table 6. By non-statistical inspection it appeared as if *Tragus berteronianus*, *Aristida *species and *Oropetium capensis *were mostly more abundant within the UCA-subhabitat. Preferences for the CA-subhabitat were shown by *Cenchrus ciliaris*, *Digitaria eriantha *and *Panicum maximum*. Those with no apparent preferences were *Brachiaria deflexa *and *Enneapogon cenchroides*, being abundant in all subhabitats. No conclusion can be drawn for *Urochloa mosambicensis*, *Bothriochloa radicans *and *Sporobolus ioclados *due to a low representation. Preferences for the RCA-subhabitat are likely to be transient in view of the expected short-term advantage that the RCA-subhabitat offers.

**Table 6:** Mean percentage contribution (on a dry mass basis) of the most abundant grass species to the total grass DM yield within the defined subhabitats.

Grass species	Exp. plot	Mean % contribution (standard error)		
		Between trees (UCA)	Under trees (CA)	Trees removed (RCA)
*Tragus berteronianus*	0 %	14.70 (7.30)	-	7.23 (4.55)
,,	10 %	8.30 (4.33)	4.30 (3.09)	5.73 (5.29)
,,	20 %	19.03 (12.97)	6.50 (3.26)	13.40 (6.95)
,,	35 %	28.57 (14.32)	9.03 (4.12)	7.03 (6.54)
,,	50 %	14.23 (7.23)	3.57 (2.51)	5.13 (4.84)
,,	75 %	11.00 (6.32)	3.60 (2.75)	5.90 (5.55)
,,	100 %	10.87 (8.76)	2.10 (2.10)	-
				
*Aristida *species	0 %	56.80 (2.59)	-	10.53 (1.43)
,,	10 %	38.23 (2.69)	12.93 (7.35)	6.23 (1.53)
,,	20 %	31.17 (4.93)	14.33 (5.80)	6.90 (3.58)
,,	35 %	8.97 (2.41)	5.23 (3.25)	8.27 (3.93)
,,	50 %	12.00 (6.89)	6.57 (3.31)	5.47 (2.39)
,,	75 %	23.37 (20.02)	4.53 (2.58)	9.37 (7.00)
,,	100 %	23.73 (7.05)	0.13 (0.13)	-
				
*Oropetium capensis*	0 %	1.93 (1.41)	-	0.13 (0.13)
,,	10 %	3.47 (1.09)	0.70 (0.60)	0.27 (0.22)
,,	20 %	6.33 (4.16)	2.17 (1.31)	0.03 (0.03)
,,	35 %	9.37 (4.27)	3.67 (1.95)	0.17 (0.17)
,,	50 %	14.17 (3.68)	3.90 (1.57)	0.10 (0.10)
,,	75 %	35.60 (15.69)	5.97 (0.85)	0.00 (0.00)
,,	100 %	21.83 (2.28)	4.93 (2.17)	-
				
*Cenchrus ciliaris*	0 %	1.67 (1.67)	-	2.10 (1.24)
,,	10 %	0.00 (0.00)	0.00 (0.00)	2.97 (1.49)
,,	20 %	0.00 (0.00)	7.63 (3.71)	1.20 (1.20)
,,	35 %	0.00 (0.00)	0.00 (0.00)	0.00 (0.00)
,,	50 %	0.00 (0.00)	4.33 (4.33)	0.00 (0.00)
,,	75 %	0.00 (0.00)	10.77 (9.21)	0.50 (0.50)
,,	100 %	0.00 (0.00)	19.63 (11.67)	-
				
*Digitaria eriantha*	0 %	0.00 (0.00)	-	1.87 (1.13)
,,	10 %	1.20 (1.20)	6.00 (3.81)	2.00 (0.91)
,,	20 %	0.00 (0.00)	6.50 (1.50)	8.17 (3.13)
,,	35 %	0.00 (0.00)	2.10 (2.10)	2.80 (0.90)
,,	50 %	0.00 (0.00)	17.60 (10.49)	8.80 (4.28)
,,	75 %	0.00 (0.00)	10.10 (3.26)	9.93 (8.52)
,,	100 %	2.37 (2.37)	40.10 (15.54)	-
				
*Panicum maximum*	0 %	0.00 (0.00)	-	6.53 (6.53)
,,	10 %	0.00 (0.00)	12.60 (12.60)	0.00 (0.00)
,,	20 %	0.00 (0.00)	0.00 (0.00)	0.00 (0.00)
,,	35 %	0.00 (0.00)	7.43 (7.43)	2.60 (2.60)
,,	50 %	0.00 (0.00)	0.00 (0.00)	0.00 (0.00)
,,	75 %	0.00 (0.00)	3.57 (3.57)	3.83 (3.83)
,,	100 %	0.00 (0.00)	0.00 (0.00)	-
				
*Brachiaraia deflexa*	0 %	4.93 (3.79)	-	15.40 (11.72)
,,	10 %	6.40 (3.13)	21.33 (11.18)	18.77 (15.40)
,,	20 %	1.17 (0.69)	15.67 (10.05)	11.87 (7.89)
,,	35 %	12.10 (6.14)	26.57 (13.32)	30.40 (19.93)
,,	50 %	25.77 (12.91)	36.60 (20.58)	35.77 (15.44)
,,	75 %	21.57 (12.27)	41.00 (21.95)	30.23 (13.99)
,,	100 %	10.60 (6.74)	26.40 (19.59)	-
				
*Enneapogon cenchroides*	0 %	16.57 (8.67)	-	47.10 (13.78)
,,	10 %	36.63 (2.47)	38.83 (13.97)	51.63 (21.41)
,,	20 %	37.57 (13.30)	42.40 (9.12)	54.20 (17.82)
,,	35 %	12.03 (6.03)	29.20 (17.05)	39.53 (16.10)
,,	50 %	26.03 (12.07)	18.77 (12.37)	42.83 (15.25)
,,	75 %	6.23 (3.83)	20.57 (12.13)	36.30 (5.18)
,,	100 %	0.60 (0.60)	6.30 (5.33)	-
				
*Bothriochloa radicans*	0 %	0.00 (0.00)	-	0.00 (0.00)
,,	10 %	0.00 (0.00)	0.00 (0.00)	9.13 (9.13)
,,	20 %	4.43 (4.43)	4.83 (4.25)	0.00 (0.00)
,,	35 %	7.37 (2.35)	14.40 (9.46)	9.23 (3.38)
,,	50 %	4.17 (2.77)	0.00 (0.00)	0.00 (0.00)
,,	75 %	2.30 (2.30)	0.00 (0.00)	1.00 (1.00)
,,	100 %	19.97 (10.38)	0.50 (0.50)	-
				
*Sporobolus ioclados*	0 %	0.00 (0.00)	-	6.63 (6.63)
,,	10 %	5.83 (5.83)	1.97 (1.82)	3.30 (2.10)
,,	20 %	0.00 (0.00)	0.00 (0.00)	1.67 (1.67)
,,	35 %	0.00 (0.00)	0.00 (0.00)	0.00 (0.00)
,,	50 %	1.33 (1.33)	0.00 (0.00)	1.97 (1.97)
,,	75 %	0.00 (0.00)	0.00 (0.00)	0.00 (0.00)
,,	100 %	0.00 (0.00)	0.00 (0.00)	-
				
*Urochloa mosambicensis*	0 %	3.40 (1.76)	-	2.40 (1.88)
,,	10 %	0.00 (0.00)	0.00 (0.00)	0.00 (0.00)
,,	20 %	0.00 (0.00)	0.00 (0.00)	2.60 (2.60)
,,	35 %	0.00 (0.00)	0.00 (0.00)	0.00 (0.00)
,,	50 %	2.30 (2.30)	0.00 (0.00)	0.00 (0.00)
,,	75 %	0.00 (0.00)	0.00 (0.00)	0.00 (0.00)
,,	100 %	0.00 (0.00)	0.00 (0.00)	-

## Discussion

The botanical composition and productivity of any mature stand of vegetation is largely determined by competition [30]. The roots of woody plants are fundamental in their competitive interactions with herbaceous plants and other woody plants. Roots determine the spatial distribution of water and nutrient uptake and can cause an increase or a decrease in resource availability [31]. This aspect was clearly illustrated within the study area [28] where it was demonstrated that the total root biomass of *C. mopane *ranged from 9760 kg ha^-1 ^to 29790 kg ha^-1 ^(mean: 17354 kg ha^-1^). Of these a mean of 19 % was in the 0–1.0 mm diameter class, and 20.3 %, 16.2 % and 44.5 % in the >1.0–5.0 mm, >5.0–10.0 mm and >10 mm diameter classes respectively. A mean of 66.1 % of all fine roots (<5.0 mm) was found within the first 400 mm of the soil [28].

A subsequent study [18] presented evidence that the roots of the *C. mopane *trees are able to utilise soil water at a matric potential lower than that of grasses (ψ < -1500 kPa). This feature, combined with high rainwater runoff losses due to a lack of a herbaceous cover, resulted in a dramatic reduction in the amount of plant available water with an increase in tree density. This enables the *C. mopane *trees to compete successfully with herbaceous plants and to prevent their establishment at high tree densities.

In view of this knowledge the observed increase in grass DM yield after the thinning of the *C. mopane *trees was expected. Indeed competitive interactions between the woody and herbaceous components of savannas, involving mainly available soil water as the primary determinant of production, have been reported world-wide (Australia: [32-35]; North America: [36-40]; southern and east Africa: [8,9,11,12,14]). While the existence of negative competition interactions between woody and herbaceous plants are thus nothing new, the results of this study is of particular significance, which relates to the magnitude and scale of the competition interaction.

The suppressive effect of the *C. mopane *trees on the grass DM yield at high tree densities is severe and for this reason the thinning of the *C. mopane *trees resulted in significant and desirable increases in grass DM yields (Figures 1 and 2). It is also worthy to note that during a wet year, like the second season, the grass DM yield differed substantially (1211%) between the extreme ends of the competition gradient (0 % versus 100 % plots), but the difference enlarged even further (2778 %) during a dry season (season 3). This substantiates the general assumption that the consequences of bush encroachment is at its worst during dry periods [9].

The gradual establishment of the strong negative relation between grass DM yield and ETTE ha^-1 ^is clearly illustrated in Figure 4. As bare soil became colonised by grasses in the plots with a low tree density, runoff of rainfall was increasingly reduced, increasing the amount of soil water available to the establishing grasses [18]. This resulted in increased differences in grass DM yield between plots at the extreme ends of the ETTE gradient. This phenomenon is clearly illustrated in Figure 1 where the grass DM yield in all the plots followed the pattern of seasonal rainfall, except in the totally cleared plot (0%) which continued to improve during the third season, while receiving less than half of the rainfall of the previous wet season (second season). At the other end of the gradient, grass DM yields differed little between years of below and above average rainfall (75 % and 100 % plots). This is typical of a human induced drought situation and not a climatic drought.

The negative curvilinear relationship between tree density and grass DM yield evident on the experimental site as a whole, as well as within the individual subhabitats, corresponds to those described for some other savanna vegetation types (e.g. [7-9], [32-34]). This relation implies that the highest grass DM yield is obtained where all the *C. mopane *trees are removed. Since grass yields under tree canopies were not significantly higher than between tree canopies, and the high yields where trees were removed are likely only temporary, it would appear that no advantageous tree-grass interactions, evident in several other savanna vegetation types (e.g. [12-15], [41-44]) occur in this vegetation type. This, at least, applies to the total grass DM yield and not to possible differences in grass species composistion.

While subhabitat differentiation did not present any advantage in respect of total grass DM yield, differences with regard to grass species composition between subhabitats were, however, present (Table 6). On evaluating these species differences, it is clear that the CA-subhabitat is important to the presence of desirable perennial grass species like *Cenchrus ciliaris*, *Digitaria eriantha *and *Panicum maximum*. These are also the species with the highest nutritional characteristics. Though these species did not constitute a large proportion of the grass species composition, they may play an important role in the total nutrition of grazing herbivores should they increase under improving management conditions.

A possible explanation for the negative reaction of forbs to tree thinning, lies in the seemingly inability of forbs to compete with establishing grasses. Thus, in those plots at the low end of the ETTE gradient, forbs were being replaced by strongly competitive grasses. The decrease of forbs can therefore be considered as a secondary consequence of the removal of *C. mopane *trees. However, it can be expected that the different forb species will react differently to competition from grasses, as well as to subhabitat changes. Thus, a proper understanding of the dynamics of forbs would necessitate an evaluation on a species basis.

The results of this study must be viewed in relation to different hypotheses of tree-grass dynamics, especially in semi-arid environments. This will invariably have an influence on the decision of the desirability to thin or clear the *C. mopane *trees for the purpose of increasing the herbaceous yield.

The terms "equilibrium" and "non-equilibrium" as used in rangelands, are points of strong debate among scientists. The central aspect of this debate is the definition of the degree to which climate or consumers (herbivores) influence vegetation. One view is that consumers reach densities that degrade environments from a previous condition of equilibrium and the other view is that the dynamics of pastoral systems are non-equilibrial and primarily dictated by variability in rainfall [45].

Higgins *et al. *[46] suggested a non-equilibrium mechanism of coexistence for savanna ecosystems. According to their model, grasses and trees coexist for a wide range of environmental conditions, and exhibit long periods of slow decline in adult tree numbers interspersed with relatively infrequent recruitment events. Recruitment is controlled by rainfall (which limits seedling establishment) and fire (which prevents recruitment into adult size classes). On the other hand, Illius and O'Connor [47] argued that the view that herbivory has little impact on climatically variable ecosystems is unjustified. They proposed an alternative model in which it is assumed that despite the apparent lack of an equilibrium, animal numbers are regulated in a density-dependent manner by the limited forage available in key resource areas which are utilized in the dry season. Their model asserts that strong equilibrial forces exist over a limited part of the system, with the animal population virtually uncoupled from resources elsewhere in the system.

While these arguments mainly relate to the causes and mechanisms according to which the woody plants increase (bush encroachment), the results of this study clearly showed that once the *C. mopane *has established, the suppression of the herbaceous layer was such that rainfall had very little effect on annual herbaceous yields in plots with high tree densities. Rainfall only played a significant role on herbaceous yields in plots where tree densities were reduced. Furthermore, the tree densities remained very stable at high tree densities with no indication, yet, of a natural process of restoration from its current encroached state.

## Conclusion

From this study it can be concluded that the grass component of the herbaceous layer, in terms of total DM yield, reacted positively to the tree thinning treatments, but forbs were negatively influenced. It is also evident that rainfall played an important role by interacting with tree density in influencing grass DM yields. Comparatively, the grass DM yields in thinned plots were substantially higher than those of the control plot during years of below average rainfall, while at high tree densities yields differed little between seasons of varying rainfall.

At high tree densities the suppressive effect of the *C. mopane *trees approach complete suppression of the grass layer. The observed curvilinear relationship between grass DM yield and ETTE ha^-1^, best described by the exponential regression equation, implies that the highest grass DM yields will be achieved when all the *C. mopane *trees are removed.

The question may then be asked if total tree clearing is the recommended option for land managers who have to deal with this problem in a practical manner. Based on some observed qualitative benefits of subhabitat differentiation by the *C. mopane *trees, with certain desirable grass species that showed a preference for the CA-subhabitat, the answer is not an unconditional yes. It is assumed that these desirable grass species would probably be lost with the complete removal of the *C. mopane *trees. From the literature it is also known that during practical tree thinning operations, re-encroachment is a common problem [28]. Through selective tree thinning, the development of a structured savanna with large trees is encouraged, and these large trees are able to suppress the establishment of new seedlings [5]. Total removal of all the *C. mopane *trees is therefore expected to be conducive to the rapid re-encroachment of the cleared area. Thinning of *C. mopane *with the exclusive objective of increasing productivity of the grass layer would thus invariably involve a compromise situation where some trees should be left for the sake of the qualitative benefits on the herbaceous layer, soil enrichment, provision of browse and stability of the ecosystem.

While the benefits of tree thinning (not total clearing) in terms of increased herbaceous yield was demonstrated in this study, the issue of cost poses a substantial limitation on the practical implementation of bush control measures in the *C. mopane *savanna vegetation. An economical evaluation of different chemical and mechanical bush control measures was beyond the scope of this study, but hopefully this study will provide essential quantitative botanical data for a thorough economical evaluation.

## Methods

### Trial layout

The study area consisted of seven, 1.17 ha plots (180 m × 65 m), thinned to differing tree densities. The plots were located next to each other on a homogeneous area of 8.2 ha. Treatments were allocated randomly to the plots. The control plot was left undisturbed (referred to as the 100 % plot), and the others thinned to the approximate equivalents of 75%, 50%, 35 %, 20 %, 10 % and 0 % (total clearing) of the tree biomass of that of the 100 % plot. The control plot was characterized by a dense stand of *C. mopane *with herbaceous plants almost completely absent.

The occurrence of dwarf growth forms of C. mopane is known to exist. In the Kruger National Park, all *C. mopane *growing on soils derived from basic material i.e. basalt, diabase/dolorite and gabbro are multi-stemmed shrubs with a mean height of 1–2 m, while *C. mopane *growing on sandy soils are usually single-stemmed and up to 5 m tall [48]. The C. mopane trees of the study area at the onset of the study had a mean height of 2.47 m (SE ± 0.052), with a mean canopy diameter of 1.68 m (SE ± 0.064). A large percentage of them was multi-stemmed. No specific information on the ages of the trees is available, but according to local inhabitants this specific dense stand of *C. mopane *trees was in existence for a number of years, though some older members can remember a time when the area was sparsely covered with trees.

Trees were randomly marked for removal during the thinning process. This ensured a fairly even spread of the remaining trees without favouring a particular tree size. The resultant thinned plots resembled the structure of naturally occurring open stands of *C. mopane*. During thinning, trees were sawn off at ground level and removed from the plot. The stumps were sprayed with a 1 % concentration of picloram and triclopyr (Tordon Super) mixed in diesel, thus ensuring that the sawn trees were killed without affecting the remaining plants. The study area was fenced to exclude grazing or browsing animals. The tree thinning was completed during the winter of 1989 and the tree densities (trees ha^-1^) were as follows: 100 % (control) plot – 2711; 75 % plot – 1978; 50 % plot – 1233; 35% plot – 744; 20 % plot – 589; 10 % plot – 300 and 0 % – 0 trees ha^-1^. The response of the herbaceous layer was studied during the three growing seasons following tree thinning.

### Rainfall

Daily rainfall data were recorded as the mean of four standard rain gauges (127 mm diameter), placed at each of the four corners of the experimental area (Figure 3).

### Quantification of the woody layer

The purpose of the survey of the woody layer was primarily aimed at obtaining some quantitative data of the leaf biomass of the remaining *C. mopan*e trees for purposes of establishing the relationship between the above ground woody and herbaceous biomass.

At the end of each growing season, normally April or May, the canopy of all rooted live *C. mopane *trees encountered in fixed transects (5 m × 180 m) located in the middle of each of the experimental plots, was measured. The measurements consisted of the following [49,50]: (i) maximum tree height, (ii) height where the maximum canopy diameter occurs, (iii) height of first leaves or potential leaf bearing stems, (iv) maximum canopy diameter, and (v) base diameter of the foliage at the height of the first leaves. The canopy volume of the trees, regardless of their shape or size, was calculated from these dimension measurements by using the volume formulas of an ellipsoid, a right circular cone, a frustum of right circular cone or a right circular cylinder. Depending on the shape of the tree, any one of these volume formulas may be used, or more likely two of them in combination. A comprehensive description of the procedure is given by Smit [50].

Leaf volume estimates (cm^3^) were calculated using the BECVOL-model (Biomass Estimates from Canopy Volume) [26,51], which is based on the quantitative description technique proposed by Smit [49,50]. It includes regression equations, developed from harvested trees, which relate the spatial canopy volume (independent variable) to the actual leaf volume (dependant variable): ln y = -4.34074 + 0.7601x, r = 0.963, P < 0.001. Spatial tree canopy volume (x) is transformed to its normal logarithmic value, while y represents the estimated leaf volume (cm^3^). The number of Evapotranspiration Tree Equivalents (ETTE) ha^-1 ^was subsequently calculated from the leaf volume estimates (1ETTE = mean leaf volume of a 1.5 m tall single-stemmed tree = 500 cm^3 ^leaf volume) [49]. Since the ETTE-values is based on estimates of actual leaf biomass it is considered a more accurate measure of potential competition of woody plants compared to simple density data (plants ha^-1^).

### Quantification of the herbaceous layer

Three subhabitats were distinguished: between tree canopies (uncanopied – UCA), under tree canopies (canopied – CA) and where trees have been removed (removed canopy – RCA). The *C. mopane *trees do not have wide spreading canopies. Closed canopies [15] are thus largely absent. The various subhabitats were consequently not considered to be purely a function of the area overspanned by the tree canopies, but also of the soil. Large scale loss of topsoil, partially retained under the trees, has led to distinctive elevated soil surface patterns. The CA and RCA subhabitats are subsequently often smaller in diameter than the immediate overstory canopy spread. Due to the relatively close proximity of the trees, as well as an extensive horizontal spread of the roots of *C. mopane *[17], the uncanopied subhabitat fell within the root zone of the trees, even at the lowest tree density.

Areas covered by the various subhabitats were determined for each of the experimental plots. Subhabitat areas, which were mostly circular in shape, were determined from two diameter measurements rectangular to each other. The area of either a fitting circle or ellipsoid was calculated [52]. Only the areas of the CA and RCA subhabitats were measured. For each experimental plot the area of the UCA subhabitat was calculated from subtracting the combined areas of the two measured subhabitats from the total area of each experimental plot (1.17 ha).

Above-ground dry matter (DM) yield of herbaceous plants within the seven tree density plots was determined at the end of each growing season, normally April or May. A harvest technique [53,54] was employed, which provided estimates of net primary production [55,56] less possible dry matter loss due to grass mortality. Losses due to grazing during the growing season were prevented by the fencing of the study area. Controlled grazing by cattle during the dormant season annually, ensured that carry-over from one season to another was low.

Grasses (species basis) and forbs (non-species basis) were harvested in quadrates (0.25 m^2^), randomly placed in each of the subhabitats. A total of 60 quadrates per experimental plot were harvested, 20 randomly allocated per subhabitat. In those plots where only 2 of the 3 defined subhabitats were represented (0 % and 100 % plots), 30 quadrates were harvested on each of the 2 represented subhabitats. Rooted herbaceous plants within each quadrate were clipped to stubble height using hand clippers. Stubble height varied from 0.1–3.0 cm, depending whether the species was tufted or not. The clipped material was dried to a constant mass (70°C) and weighed.

### Data analyses

In testing for treatment effects, care was taken to avoid the use of pseudo-replications [57]. Relations between tree leaf biomass (dry basis) and the DM yield of herbaceous plants (grasses and forbs) were established using regression analyses [58,59]. For the determination of differences in trend of grass DM yield between habitats and subhabitats, the total grass DM yield within the various habitats and subhabitats of the experimental plots after each successive season (x-axis), was subjected to the fitting of polynomials. Polynomials (linear, quadratic or cubic) with the best fit were selected and paired combinations of these selected polynomials were subsequently tested for contrasts on the x-axis (tree density) using the procedures of Groeneveld [29].

## List of abbreviations

BECVOL – Biomass Estimates from Canopy Volume

CA – Canopied (under tree canopies)

DM – Dry mass

ETTE – Evapotranspiration Tree Equivalents (1 ETTE = mean leaf volume of a 1.5 m single-stemmed tree = 500 cm^3 ^leaf volume)

RCA – Removed Canopy (where trees were removed)

UCA – Uncanopied (between tree canopies)

## Authors' contributions

All aspects of the study were conducted by the author with assistance as indicated under Acknowledgements.
